# Anaphylactic reaction following administration of nose drops containing benzalkonium chloride

**DOI:** 10.1186/1746-160X-8-29

**Published:** 2012-10-18

**Authors:** Elke Mezger, Olaf Wendler, Susanne Mayr, Alessandro Bozzato

**Affiliations:** 1 Department of Otorhinolaryngology—Head and Neck Surgery, University of Erlangen, Waldstraße 1, Erlangen 91054, Germany

**Keywords:** Allergy, Anaphylactic reaction, Benzalkonium chloride

## Abstract

We describe a case of anaphylactic reaction in a 46-year-old female post application of decongestant nose drops containing benzalkonium chloride (BAC). With some latency, the patient complained of cough, dyspnea, sensation of heat, croakiness and pruritus. Since she showed all of these symptoms, typical of an anaphylactic reaction, we proceeded some weeks later with a prick test with solutions containing BAC, a cationic surfactant commonly used as an antibacterial preservative in many medical solutions. The prick test was positive, confirming the assumption of a hypersensitive reaction to BAC.

## Background

This is the first case described in the literature of an immediate-type (type I) allergic reaction to nose drops containing benzalkonium chloride. Benzalkonium chloride (N-alkyl-N-benzyl-N,N-dimethyl ammonium chloride) is a quaternary ammonium compound used, for instance, as a preservative agent in topical medications (eye and nose drops).

## Case presentation

A 46-year-old female patient presented in our outpatient department with an existing nasal respiration obstruction she had been experiencing for about 3 months with right-side preponderance, severe right-side facial pain and a subjectively reduced sense of smell. Surgical restoration of the right maxillary sinus was considered due to the ineffectiveness of the oral antibiotic and steroid treatments carried out to date. Six years earlier, the patient had undergone an endonasal septal deviation pansinus operation with septoplasty in the treatment of chronic ethmoid and maxillary sinusitis. In the years between this procedure and recurrence of the symptoms described above, the patient was otherwise free of symptoms, undergoing only various dental procedures.

### Examination and findings

A rhinoscopic examination initially revealed nearly complete nasal obstruction by an inflammatory hyperplasia of the inferior turbinate on the right side and unremarkable findings on the left side. Further endoscopic examination revealed inflammatory narrowing of the right middle nasal meatus, with plentiful putrid secretion and polypoid mucosa in the area of the right ethmoid bone. The left middle nasal meatus was open and the septum position was median postoperatively. The remaining ENT findings were normal, in keeping with the patient's age. The findings from the computed tomography of the nasal sinuses done one week previously were of right-side sinusitis with a broad area of mucosal swelling around the fenestrated maxillary sinus as well as of the nasal cavity and the right-side portion of the sphenoid sinus and ethmoid cells. The images showed open nasal sinuses on the left side. The results of a prick test for fungal allergens performed in 2004 prick were a double positive reaction to Aspergillus fumigatus and a simple positive reaction to Cladosporium herbarum. There is otherwise no known history of further allergies.

### Diagnostics

A rhinomanometric examination confirmed the right-side nasal obstruction, with normal findings for the left side. The olfactory test result was normosmic. Diagnostics continued with mucosal detumescence by means of spray application of Otriven® (from Novartis, containing 100 ml,), then high insertion. It was then possible to aspirate the putrid secretion and explore the maxillary sinus. Chunky gray material was removed that suggested an aspergilloma. However, after about 20 to 30 min, the patient then reported increasing massive bilateral obstruction of the nose and a strong sense of heat in the head. She also complained of itching in the throat, hoarseness, edematous swelling of the uvula and soft palate, and vesicles on the posterior pharyngeal wall.

A synoptic analysis of the symptoms led us to suspect an anaphylactic reaction to components of Otriven®.

## Therapy

Acute treatment of the anaphylactic reaction comprised Soludecortin H (250 mg iv.), one ampoule of Tavegil® (clemastine fumarate), one ampoule of Ranitic® (ranitidine), oxygen through a nasal tube (3 l/min) and volume supplementation with Jonosteril® infusions (1 l/hour). The patient was hospitalized for monitoring.

## Course

The patient's condition returned to normal within four hours. She continued to receive nasal irrigation and a nasal emulsion formulated in our hospital that contained no preservatives whatever. It was possible to discharge the patient to outpatient care after two days. After four weeks she presented once again for an ENT monitoring examination. At this time she was completely free of symptoms, i.e. was not experiencing nasal obstruction and/or facial pain. The endoscopic examination showed an unremarkable lower and middle nasal turbinate on the right side, an unobstructed view into the maxillary sinus ostium and free ascension to the frontal sinus.

Six weeks after the anaphylactic event, a prick test was carried out with three different concentrations of benzalkonium chloride (0.01% solution, 0.1% solution, 0.5% solution, each dissolved in H2O). 0.1% elicited a mild reaction (simple positive), 0.5% a very strong reaction (triple positive), including negative control with NaCl and positive control with histamine hydrochloride (10 mg/ml). A prick test was conducted with the same solutions in a control group of 15 volunteers, with negative results in all cases. The suspected diagnosis of allergy to benzalkonium chloride was therefore confirmed.

## Discussion

This is the first case described in the literature of an immediate-type (type I) allergic reaction to nose drops containing benzalkonium chloride. Benzalkonium chloride (N-alkyl-N-benzyl-N,N-dimethyl ammonium chloride) is a quaternary ammonium compound used, for instance, as a preservative agent in topical medications (eye and nose drops). It is also used in disinfectants, medicinal gargle solutions, throat lozenges, adhesive bandages, as an algicide in swimming pools and in medicinal cosmetics; it is also used as an industrial chemical in dye synthesis and metallurgy, in agriculture and the textile industry. Numerous cases of allergic reactions to this quaternary ammonium compound have been reported [1], also in the context of type I reactions to antiasthmatic sprays [2], eye drops [3] and ear drops [4] containing benzalkonium chloride. The symptoms ranged from paradoxical bronchoconstriction to anaphylactic reaction and angioneurotic edema [3]. Occurrence of toxic contact dermatitis in a type IV reaction [5] has also been described. A property causing direct damage to cilia has also been reported, which must be kept in mind above all when the agents are used on mucosa with ciliated epithelia such as those of the nose or upper respiratory tract [6]. This is discussed mainly in connection with a drug rhinitis, which is by definition not caused by an allergy, but rather by a directly harmful effect of the agent that manifests after regular use [7]. It has, however, been demonstrated in various animal studies that benzalkonium chloride results in increases of total IgE and IgG1 in the blood, mediated by TH2 lymphocytes, and thus causes sensitization [8]. It can therefore be assumed that the reaction to benzalkonium chloride was presumably an IgE-mediated, type 1 reaction, although non-specific histamine release from mast cells is also being discussed [2]. The occurrence of toxic contact dermatitis associated with benzalkonium chloride is presumably due to activation of allergen-specific cytotoxic T cells. Various diagnostic approaches are available to confirm a benzalkonium chloride allergy. Determination of antibodies, specifically total IgE and specific IgE, is a method commonly practiced in allergy diagnostics. Application of this method is, however, not feasible in the case of benzalkonium chloride since there is no specific IgE to benzalkonium chloride. A rise in total IgE may indeed be indicative of an atopic disposition, but it is not specific enough to be used as a diagnostic tool, since it is not conclusive and an allergy may also be present in the absence of an increase. As described above, benzalkonium chloride also leads to an increase of IgG1. Determination of this antibody is not useful because it can be detected in sera from both healthy and atopic individuals. It also merely reflects exposure to an allergen. There is also the possibility, for allergies associated with mast cells, of determining mast cell tryptase, whereby it is important to determine the initial value as a basis for comparison, which is often not possible. Secondary diagnostics also include the basophil stimulation test, which can be interpreted as an indirect measure of cell-bound IgE and, when other stimuli are applied, is also indicative of IgE-independent reactivity of the leukocytes involved. Implementation of this test requires a concentration of blood leukocytes, making it technically demanding to a certain degree. Since benzalkonium chloride can also cause type IV reactions, a further method that can be applied is the lymphocyte transformation test, in which lymphocyte cultures are exposed to antigen-presenting cells and the suspected allergen, whereby incorporation of H3-thymidine serves as the measure of lymphocyte transformation. This test cannot, however, distinguish between a physiological response to an antigen and an allergic reaction and also poses high technical requirements. Skin tests such as the prick and intracutaneous tests are well-established in allergy diagnostics and are particularly well-suited to identify type I reactions. We also decided to employ these methods, to which end we created a test series of different solutions containing benzalkonium chloride in different concentrations. We were able to confirm the allergy in this patient by employing this method.

## Conclusions

Medical application of decongestant nose drops should also take into consideration the rare, but possible, allergic reaction to benzalkonium chloride as described above. Questions about previous allergic reactions or symptoms in reaction to similar drugs must therefore be included in the medical history interview and, if there is any doubt, clarification should be based on a prior allergy test. In case of a positive result, patients must be carefully informed and instructed concerning strict avoidance of substances containing the preservative agent and the need to contact a physician without delay if allergic symptoms occur. A further alternative for patients with rhinitis symptoms is use of decongestant nose drops that contain no preservatives.

## Consent

We state that the patient has given her consent for the Case report to be published.

## Competing interests

We state that there are no financial disclosures and that there is no conflict of interest.

## Authors’ contributions

EM, acquisition of data, analysis and interpretation of data, preparation of manuscript. OW, drafting the manuscript or revising it critically. SM, drafting the manuscript or revising it critically. AB, conception and design. All authors read and approved the final manuscript.
